# Investigation of Nutritional Behaviors in the First and Second Trimesters in Pregnant Women Referring to Clinics in Hamadan, Iran, in 2013

**DOI:** 10.5539/gjhs.v8n9p261

**Published:** 2016-01-31

**Authors:** Seyedeh Zahra Masoumi, Parisa Parsa, Farideh Kazemi, Ali Reza Soltanian, Gissoo Dadvand, Shabnam Habib

**Affiliations:** 1 Research Center for Child & Maternity Care, Midwifery Department, School of Nursing and Midwifery, Hamadan University of Medical Sciences, Hamadan, Iran; 2 Parisa Parsa, Research Center for Chronic Disease (home) Care, Department of Mother and Child Health, Hamadan University of Medical Sciences, Hamadan, Iran; 3 Department of Midwifery & Reproductive Health, School of Nursing & Midwifery, Shahid Beheshti University of Medical Sciences, Tehran, Iran; 4 Modeling of Noncommunicable Diseases Research Center and Department of Biostatistics, School of Public Health, Hamadan University of Medical Sciences, Hamadan, Iran; 5 Department of Midwifery, Hamadan University of Medical Sciences, Hamadan, Iran

**Keywords:** pregnant women, nutritional behaviors, nutrition

## Abstract

**Background:**

Pregnancy is a particular period in women’s life that is accompanied by an increase in nutritional needs. Having a normal pregnancy period and successful pregnancy outcomes depends on the intake of sufficient amount of food. The present study aimed to determine nutritional behaviors in pregnant women in the first and second trimesters referring to clinics in Hamadan, Iran.

**Methods:**

This cross-sectional study was conducted on 170 women referred to health and treatment centers of Hamadan in 2013. Among Hamadan’s health and treatment centers, 10 were selected as the research setting through cluster sampling. Then, the pregnant women at 8-24 weeks of gestation were randomly entered into the study. The data were collected using nutritional behaviors questionnaire in three months. Accordingly, mean scores below 50, between 50 and 75, and above 75 were considered as weak, moderate, and perfect nutritional behaviors, respectively. The data were analyzed using the SPSS statistical software, version 21 and P<0.05 was considered as statistically significant.

**Results:**

The results showed that more than three fourths of the study participants had moderate nutritional behaviors. Insufficient intake of fruit, vegetables, and cereals was detected in 80.6%, 54.1%, and 47.1% of the participants, respectively. Besides, 30% of the women had not used folic acid supplement during their pregnancy period or were not aware of its necessity. The results of multivariate analysis indicated that age was only significantly associated with pregnant women’s score of nutritional behaviors (P=0.010). Additionally, no significant relationship was found between the women’s nutritional behaviors and their strategies for elimination of common pregnancy complications, such as constipation, heartburn, urinary tract infection, and anemia.

**Discussion and Conclusion:**

Considering the participants’ moderate nutritional behaviors, health and treatment centers are recommended to provide the necessary training for improving pregnant women’s nutritional behaviors and supervise and follow their execution and evaluation.

## 1. Introduction

Pregnancy is considered as one of the most important and the riskiest periods of women’s life. Mother’s health in this period not only affects her quality of life, but it also plays a role in the health of her fetus and future generations (Gomez et al., 2013). Pregnancy is among the particular periods of life that is accompanied by an increase in nutritional needs (Verbeke, & De Bourdeaudhuij, 2007; Wu, Bazer, Cudd, Meininger, & Spencer, 2004). Besides, having a normal pregnancy period and successful pregnancy outcomes depends on the intake of sufficient amount of food (Verbeke, & De Bourdeaudhuij, 2007). The importance of nutrition during pregnancy with regard to pregnancy outcome has long been acknowledged. This importance has only been further emphasized by the recent changes in food quality and availability, lifestyle changes and a new understanding of fetal programming’s effects on adult outcomes (Mparmpakas et al., 2013). Food diet is important for development and differentiation of organs within the first trimester and for overall fetal development and brain development in the following trimesters (Rifas-Shiman, Rich-Edwards, Kleinman, Oken, & Gillman, 2009). In addition to sufficient nutrition, balance between nutrients is of particular importance (Cunningham et al., 2009). Unfavorable maternal, neonatal and child health outcomes remain prevalent in developing countries (Khan, Wojdyla, Say, Gulmezoglu & Van Look, 2006) and inappropriate balance in eating diet can have long-term consequences for both mother and her infant (Fowles, 2004). In fact, mother’s diet during pregnancy can affect the length of pregnancy, fetal growth, birth defects, infant’s cognitive growth and obesity (Rifas-Shiman et al., 2009; Sebire, Jolly, Harris, Regan, & Robinson, 2001).

Moreover, numerous studies report a role for modifiable maternal nutrition practices in pregnancy-induced hypertension (PIH), hemorrhage, severe anemia, obstructed labor, infections, unsafe abortions and their subsequent complications (Melah et al., 2007; Muleta, Rasmussen, & Kiserud, 2010; Rush, 2000). For example, women with calcium deficiency have a greater risk of PIH (Ritchie, & King, 2000; Villar & Belizan, 2000) while supplementation is associated with a 50% reduction in the risk of pre-eclampsia (Trumbo, & Ellwood, 2007). Anemia, and especially severe anemia, are associated with an increased risk of maternal mortality (Rush, 2000; Villar et al., 2003). Micronutrient deficiencies during pregnancy as well as inadequate weight gain have significant implications for neonatal and infant outcomes, including preterm delivery, low birth weight (LBW) and birth defects (Abu-Saad & Fraser, 2010). On the other hand, correct and sufficient nutrition supplies fetal growth, creates resources in the fetus, and developing mineral resources in the mother’s body that is one of the main criteria of successful breastfeeding within the first 6 months after delivery (Mirmolaei, Moshrefi, Kazemnejad, Farivar & Morteza, 2010).

Despite the importance of proper nutrition during pregnancy, a large number of pregnant women do not fulfill nutritional recommendations, especially about fruits, vegetables, cereals, folate, and iron (Nash, Gilliland, Evers, Wilk & Campbell, 2013). This might be attributed to physical and physiological changes during pregnancy, which cause problems in food intake through changing the sense of taste, loss of appetite, edema, and pica (George, Hanss-Nuss, Milani, & Freeland-Graves, 2005). Another reason can be pregnant women’s low nutritional knowledge that is a determining factor for unhealthy nutritional behaviors (Fallah, Pourabbas, Delpisheh, Veisani & Shadnoush, 2013). Studies performed in Iran have also confirmed pregnant women’s low level of knowledge about healthy nutrition during this period (Kamalifard, Mohammad-Alizade-Charandabi, Ebrahimi-Mamegani, Asghari-Jafarabadi, & Omidi, 2012; Sajjadi, Bakhtiari, & Haji Ahmadi, 2007) and their inappropriate nutritional status (Abedini, Ahmari Tehran, Gaini, & Khorami Rad, 2011).

Development of healthy behaviors at all stages of life is one of the main objectives of “healthy people 2020” (Lyons, 2014). In this regard, behavior modification is one the risk reducing strategies, and planning for behavior change requires knowledge about the problems related to unhealthy behaviors (Mohammad Alizadeh Charandabi, Kamali Fard, Ebrahimi Mamqani, Asghari Jafarabadi, & Omidi, 2012). Thus, in order to execute effective interventions for improving pregnant women’s nutritional function, first the population’s nutritional function should be assessed, so that the problems can be solved by identification of the areas requiring intervention and provision of the necessary training. Hence, the present study aims to determine nutritional behaviors among the pregnant women in the first and second trimesters.

## 2. Methods

### 2.1 Study Design

Cross sectional, descriptive analytic study.

### 2.2 Participant

This study was conducted on 170 pregnant women referred to health and treatment centers of Hamadan in 2013.

### 2.3 Data Collection

Among Hamadan’s health and treatment centers, 10 were selected as the research setting using cluster sampling. Then, the pregnant women at 8-24 weeks of gestation who had referred to these clinics and met the inclusion criteria were enrolled into the research. The inclusion criteria of the study were not suffering from chronic disorders (gastrointestinal disorders, diabetes, cardiovascular diseases, hypertension, and cancer) with specific diets, not having had pregnancy complications, such as bleeding, during the first trimester, not suffering from preeclampsia and overt diabetes, not using alcohol or drugs, and not having the history of unilateral oophorectomy and myomectomy. On the other hand, the exclusion criteria of the study were not completing the study questionnaire and suffering from pregnancy complications during the study.

### 2.4 Questionnaires

The study data were collected using a questionnaire including two parts. The first section involved socio-demographic information, including age, height, weight, education level, occupation, husband’s education level, income level, and length of marriage. The second part, on the other hand, included awareness of nutritional behaviors questionnaire. This questionnaire was designed by Mohammad Alizadeh et al. in 2012 and its reliability and validity were approved. The data collection instrument contained 16 socio-demographic questions and 21 items for assessment of nutritional behaviors. The first part and some questions related to food proportions in the second part were completed by the researcher, while the rest of the items were filled by the pregnant women. Besides, the final five questions in the second section dealt with proportion of food intake from different food groups and were completed by the researcher through computation of food proportions using Food Frequency Questionnaire (FFQ). In order to determine the validity of the questionnaire, it was given to 9 faculty members (5 midwives, 3 nutritionists, and 1 gynecologist) and the necessary modifications were applied. In addition, to determine the reliability of the questionnaire, it was given to 15 pregnant women who were eligible to take part in the study with a 2 week interval. The validity and reliability of this questionnaire was previously assessed and confirmed in other studies (Mohammad Alizadeh Charandabi et al., 2012). And in our study its internal consistency was approved by Cronbach’s alpha coefficient (α=0. 87)

The study data were collected in three months. The items related to nutritional behaviors had 2-6 options and the participants had to choose one. After all, the score of nutritional behavior was calculated by dividing the scores obtained from all the items by the total number of items. Accordingly, scores below 50, between 50 and 75, and above 75 were considered as weak, moderate, and perfect nutritional behaviors, respectively.

### 2.5 Statistical Analysis

All data analyses were performed using the SPSS statistical software, version 21. Chi-square, independent t-test, one-way ANOVA, and multivariate regression analysis were used to determine the relationship between nutritional behaviors and variables, such as Body Mass Index (BMI) and protein consumption. P<0.05 was considered as statistically significant.

## 3. Results

### 3.1 Demographic Characteristics

The study participants’ age ranged from 16 to 48 years, with the mean age of 27.5 years.

### 3.2 Survey of Nutritional Behavior

**Figure 1:**
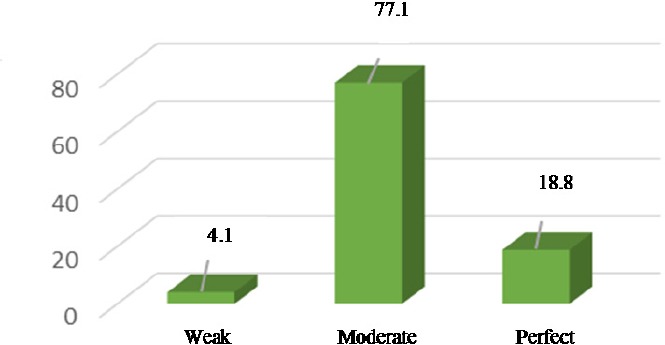
Pregnant women’s nutritional behavior status

The study participants’ age ranged from 16 to 48 years, with the mean age of 27.5 years. The mean score of nutritional behavior was 67.78±9.32, ranging from 41.18 to 94.12. Thus, more than three fourths of the study participants had moderate nutritional behaviors (Figure 1). Besides, a significant relationship was observed between age and nutritional behavior score (P=0. 007). According to the results, the individuals > 36 years old gained higher nutritional behavior scores compared to those below 36 years of age. Moreover, the prime-gravid women had better nutritional status compared to the multiparous ones. Comparison of various BMI groups also showed that increase in BMI was accompanied by an increase in nutritional score. Furthermore, the women with an academic education and those whose husbands had an academic education got higher nutritional behavior scores compared to those without academic degrees. Homemaker women and those with the income level of > 5 000 000 Rial per month also obtained higher nutritional behavior scores compared to the working women and those with below 5 000 000 Rial incomes per month. Except for age, none of the variables were associated with the mean score of nutritional behaviors (Table 1).

**Table 1:** Pregnant women’s socio-demographic features and their relationship with the mean score of nutritional behaviors in the first and second trimesters

Variable	Number (%)	Mean + SD	P-value
Age (years)			
< 36	162 (95.3)	67.35+9.09	0.007
> 36	8 (4.7)	76.47+10.18	

Number of pregnancies			
1	73 (42.9)	69.13+8.97	0.100
> 2	97 (57.1)	66.76+9.49	

BMI			
< 19.8	7 (4.1)	66.80+12.67	†0.830
19.8-24	62 (36.5)	67.12+9.12	
25-29	68 (40)	67.94+10.24	
> 29	33 (19.4)	68.89+6.93	

Woman’s education level			
Non-academic	128 (75.3)	67.5+9.23	0.610
Academic	42 (24.7)	68.41+9.68	

Husband’s education level			
Non-academic	132 (77.6)	67.51+8.99	0.480
Academic	38 (22.4)	68.73+10.44	

Woman’s occupation			
Employee	27 (15.9)	67.19+9.00	0.058
Homemaker	143 (84.1)	70.91+10.48	

Income level			
< 5 000 000 Rial per month	78 (45.9)	66.43+9.38	0.120
> 5 000 000 Rial per month	92 (54.1)	68.68+9.21	

Length of marriage			
Less than 37 months	79 (47.0)	67.87+9.66	0.950
37 months and above	89 (53.0)	67.94+8.90	

† One-way ANOVA, the rest: t-test.

In order to control the probable confounding variables, age, occupation, number of pregnancies, and income level with P < 0.2 in univariate analysis were entered into the multivariate regression model. The results revealed that age was only significantly associated with the pregnant women’s mean score of nutritional behaviors. Accordingly, as age decreased, the mean score of nutritional behavior decreased, as well (Table 2).

**Table 2:** Predictors of mean score of nutritional behavior in the first and second trimester (results of multivariate regression analysis)

Features	Number	β (95% CI*)	P-value
Age			
< 36	8	Reference	0.010
> 36	162	-0.197 (-15.19, -2.07)	

Number of pregnancies				
1	73	Reference	
> 2	97	-0.117 (-4.99, 0.60)	

Occupation				
Homemaker	143	Reference	0.080
Employee	27	0.132 (-0.45, 7.19)	

Monthly income				
> 5 000 000 Rial	92	Reference	0.300
< 5 000 000 Rial	78	-0.078 (-4.37, 1.39)		

Constant		77.33 (70.80, 83.86)	

* Confidence interval.

As Table 3 depicts, the amount of food intake had increased in the majority of the participants (70.6%). However, 40.7% of the women showed a decreased amount of fruit intake or had no accurate information in this regard. Also, almost half of the women (48.8%) showed the reduced amount of vegetable intake or had no idea in this respect. On the other hand, intake of meat and beans (71.8%) and milk or yogurt (75.9%) had increased in three fourths of the study participants. Furthermore, nearly 30% of the women did not use folic acid or had no information in this regard.

**Table 3:** Changes in the pregnant women’s nutritional behaviors in the first and second trimesters (percentage)

Questions	I don’t know	It has decreased	It has increased	Total
Has your amount of food intake changed since you have become pregnant?	16.5	12.9	70.6	100
Has your amount of fruit intake changed since you have become pregnant?	8.2	32.5	59.3	100
Has your amount of vegetable intake changed since you have become pregnant?	34.7	14.1	51.2	100
Has your amount of meat and beans intake changed since you have become pregnant?	14.1	14.1	71.8	100
How many glasses of milk or yogurt are you consuming daily since you have become pregnant?	9.4	14.7	75.9	100
Are you using folic acid during pregnancy?	20.0	9.4	70.6	100

**Figure 2:**
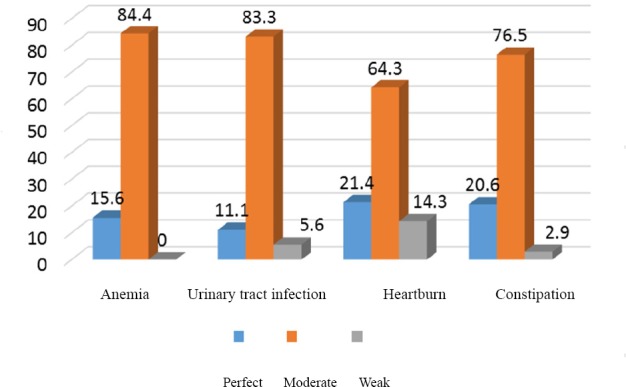
Pregnant women’s nutritional behaviors while facing common pregnancy complications

The study results revealed no significant relationship between the types of nutritional measures in common pregnancy complications and nutritional behaviors followed to eliminate those complications. Based on Figure 2, the majority of the participants who took a measure to eliminate their problems had moderate nutritional behaviors. Moreover, 78.3% of the participants showed weak nutritional behaviors in preventing constipation, 62 ones in preventing heartburn, 2.61% in preventing anemia, and 3.81% (N=12) in preventing urinary tract infection (Table 4).

**Figure 3:**
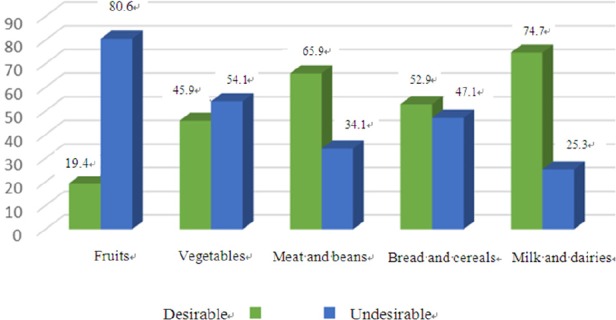
Consumption of different food groups compared to the standard recommended amount

According to Figure 3, insufficient intake of fruits, vegetables, and bread and cereals were observed in 80.6%, 54.1%, and 47.1% of the participants, respectively. However, the amount of meat and bean intake was desirable in more than two thirds of the participants. Also, 74.7% of the women showed desirable intake of milk and dairy products.

**Table 4:** The relationship between the measures taken for elimination of common pregnancy complications and the participants’ nutritional behaviors

Nutritional measures in the participants with pregnancy complications	Number (percent)	Nutritional behavior			

		Weak	Moderate	Perfect	P-value
Measures for constipation					
Yes	32 (58.2)	0	84.4	15.6	0.690
No	23 (41.8)	4.3	78.3	17.4	

Measures for heartburn					
Yes	18 (48.6)	5.6	83.3	11.1	0.790
No	19 (51.4)	0	89.5	10.5	

Measures for dysuria					
Yes	14 (51.9)	14.3	64.3	21.4	0.550
No	13 (48.1)	0	84.6	15.4	

Measures for anemia					
Yes	136 (80.0)	2.9	76.5	20.6	0.170
No	34 (20.0)	8.8	79.4	11.8	

## 4. Discussion

The findings of the present study showed that more than three fourths of the participants had moderate nutritional behaviors. This was in contrast to the results of the study by Mohammad Alizadeh et al. (Mohammad Alizadeh Charandabi et al., 2012), showing weak and moderate nutritional behaviors in half of the study participants. This difference might be due to the study population’s gestational age. Mohammad Alizadeh’s study was performed on the women in the first trimester of pregnancy in which, nausea and vomiting might affect nutritional behaviors.

Although the amount of food intake had increased among the current study participants, half of the women showed an undesirable amount of fruits and vegetables intake. In spite of the approved effects of folic acid in prevention of neural tube defects, intake of this supplement was also undesirable among 30% of the participants. Similar to our study, Bojar et al. performed a research in Poland and indicated that the amount of food intake had increased among the pregnant women compared to before pregnancy (Bojar, Wdowiak, Humeniuk, & Blaziak, 2006). In the study conducted by Verbeke et al. in Belgium in 2003 (Verbeke, & De Bourdeaudhuij, 2007), the amount of raw vegetable intake was undesirable, which was consistent with our study results, but the amount of fruit intake had increased during pregnancy. Low intake of fruits, vegetables, and folic acid in the present study might be attributed to not allocating sufficient time to training pregnant women in healthcare centers, private clinics, and mass media, resulting in pregnant women’s low knowledge level in this regard.

In the current study, almost three fourths of the participants consumed desirable amounts of meat and beans, which is in agreement with the findings of the study by and Verbeke et al. in Belgium in 2003 (Verbeke, & De Bourdeaudhuij, 2007). On the contrary, Mohammad Alizadeh et al. (Mohammad Alizadeh Charandabi et al., 2012) and Agrahar-Murugkar et al. (Agrahar-Murugkar & Pal, 2004) demonstrated in their studies that the amount of meat and bean intake was less than the recommended amount based on the food pyramid. In general, consumption of meat and beans depends on a family’s income level and purchasing power. Family’s eating habits may also play a role in this respect. In fact, an increase in the proportion of this food group in a family might decrease that of other food groups.

Similar to meat and beans, consumption of milk and dairy products was in an optimum status in our study. This was in contrast to the study performed by Mohammad Alizadeh et al. (Mohammad Alizadeh Charandabi et al., 2012). This difference might be due to the study populations’ culture or gestational age.

In the present study, the results of multivariate regression analysis revealed that age was only significantly associated with the mean score of nutritional behaviors. The individuals at the age of 36 years and above probably follow better nutritional behaviors compared to those below 36 years of age because of higher ages and high-risk pregnancies. Moreover, according to Sichert-Hellert et al. (Sichert-Hellert et al., 2011), an increase in age led to an increase in nutritional knowledge.

The findings of the present study showed that more than 80% of the participants had weak and moderate behaviors in controlling heartburn and constipation. This measure was obtained as 80% regarding urinary tract infection and anemia. Mohammad Alizadeh et al. (Mohammad Alizadeh Charandabi et al., 2012) also came to similar results regarding heartburn and constipation, but reported more than 80% for urinary tract infection and anemia. One reason for not taking correct nutritional behaviors while encountering common pregnancy complications might be pregnant women’s lack of knowledge in this respect, which is mainly related to service providers. Arrish et al. conducted a study in Australia in 2014 and stated that midwives did not have enough knowledge about nutrition during pregnancy, which was associated with insufficient training during their B.Sc. and M.Sc. courses (Arrish, Yeatman, & Williamson, 2014). Nutritional training in pregnancy can reduce common pregnancy complications to a great extent. Of course, this depends on service providers having sufficient knowledge and allocating enough time for training pregnant women.

## 5. Conclusion

Nutrition is an inseparable part of human life and is of utmost importance because a lot of diseases and problems are rooted in inappropriate nutritional behaviors. Despite the importance of nutrition during pregnancy, nutritional problems are still observed in this vulnerable group. According to the findings of the present study, pregnant women had moderate nutritional behaviors. Therefore, health and treatment centers are recommended to provide the necessary training for improving pregnant women’s nutritional behaviors and supervise and follow their execution and evaluation. In order to provide pregnant women with correct training, service providers should also have sufficient knowledge in this regard. Moreover, training healthy nutritional behaviors should be started from childhood and school age and, at the same time, families are required to enhance their knowledge in this respect. Furthermore, since nutrition is affected by the society’s food culture, policymakers have to take measures to change unhealthy nutritional behaviors in the society, particularly among pregnant women who are responsible for the next generation, so as to create a healthy, dynamic, and constructive generation. The limitation of this study was small sample size. And conducted a study with a larger sample is recommended.
